# Pulsed Electric Field Ablation for Advanced Lung and Oligometastatic Disease: A Retrospective Study of 32 Consecutive Patients in a Community Hospital Setting

**DOI:** 10.3390/cancers18091459

**Published:** 2026-05-01

**Authors:** Varun Roperia, Justin Thomas

**Affiliations:** 1 Internal Medicine, Eisenhower Health, Rancho Mirage, CA 92270, USA; vroperiamd@gmail.com; 2 Interventional Pulmonology and Critical Care, Eisenhower Health, Rancho Mirage, CA 92270, USA

**Keywords:** tumor ablation, pulsed electric field, abscopal response, lung cancer

## Abstract

Background/Objectives: Pulsed Electric Field (PEF) therapy is a non-thermal ablation technique that induces immunogenic cell death through high-voltage, short-duration electrical pulses. This may enhance antitumor immunity by releasing intact tumor antigens and potentially generating abscopal effects. We report early outcomes in 32 patients with primary lung cancer or lung oligometastases treated with PEF at a community hospital, with a median (IQR) follow-up of 180.5 (158–207) days. Methods: This retrospective study collected demographics, cancer type, treatment response, and outcomes for patients undergoing PEF ablation. Tumor response was assessed using Sum of Longest Dimensions per RECIST 1.1 to classify progressive disease, stable disease, partial response, or complete response. Volumetric changes were additionally analyzed using RECIST 1.1 percentage thresholds applied to change in volume. Results: At initial 3-month follow-up, 26 of 32 patients demonstrated stable disease, partial response, or complete response, suggesting an 81.25% disease control rate/clinical benefit rate among this cohort. Among patients with Stage III–IV disease, 27.6% (8/29) showed radiographic evidence of a possible abscopal response. At 6 months, 24 of 32 patients remained alive and evaluable, with 62.5% (20/32) maintaining stable disease, partial response, or complete response. Conclusions: Despite patients having progressive disease on systemic therapy before PEF, early outcomes post-ablation suggest favorable local control and potential immunologic benefit. Patients with early-stage disease not receiving systemic therapy also showed excellent local response. Patients tolerated therapy very well. Clinical benefit was observed in 81.25% of patients at 3 months and 62.5% at 6 months, with radiographic evidence of possible abscopal responses in 27.6% of advanced-stage patients, supporting further exploration of the immunogenic potential of PEF demonstrated in preclinical and emerging clinical studies.

## Simple Summary

Pulsed Electric Field (PEF) ablation is a novel, non-thermal technique that destroys tumor cells using brief high-voltage electrical pulses and may stimulate an immune response by releasing intact tumor antigens. While early trials have shown that PEF is feasible and safe, its real-world clinical performance in lung cancer remains in need of further research. In this retrospective study, we report early outcomes from 32 patients with primary lung cancer or lung oligometastases treated with PEF ablation in a community hospital setting. Many patients had disease progression despite systemic therapy prior to ablation. At three months, 81.25% of patients demonstrated stable disease, partial response, or complete response; 62.5% maintained this benefit at six months, including both patients with complete response. Some patients with advanced disease also showed radiographic evidence of abscopal responses, possibly suggesting immune-mediated effects beyond the treated tumor. These findings offer real-world evidence data of PEF in a community hospital setting, aim to inform future clinical research via provision of hypothesis-generating data, and help define the potential role of PEF both as a stand-alone and as part of an immunomodulatory treatment strategy in thoracic oncology.

## 1. Introduction

Despite substantial progress in systemic therapies, lung cancer remains the leading cause of cancer mortality and lung metastasis remain linked to poor prognosis across multiple cancers [1,2]. A significant unmet need persists for patients with advanced lung cancer and oligometastatic disease involving the lungs—particularly those with progression despite front-line treatment. Many of these patients are poor candidates for surgical resection or conventional thermal ablative modalities due to tumor location, comorbidities, or prior therapies.

Pulsed Electric Field (PEF) therapy is an emerging, non-thermal ablation modality that delivers high-voltage, microsecond-duration electrical pulses to targeted tissues, locally altering cellular transmembrane potentials, disrupting cell homeostasis, and subsequently triggering signaling pathways that lead to regulated cell death. In contrast to conventional thermal ablation methods such as radiofrequency or microwave ablation, PEF primarily causes cell death via necroptosis, apoptosis, and pyroptosis instead of necrosis-dominant cell death and does not rely on heat generation. This allows for precise and tissue-selective energy delivery while preserving the extracellular matrix, vascular structures, and surrounding normal parenchyma [3]. These characteristics make PEF particularly suitable for treating tumors located near critical structures where conventional thermal ablation would be contraindicated, with early clinical evidence proving consistent with this safety profile [4].

Beyond direct cytotoxicity, accumulating preclinical and early clinical evidence suggests that PEF therapy exerts meaningful immunomodulatory effects within the tumor microenvironment [5]. PEF-induced cell death is characteristically immunogenic, associated with the release of damage-associated molecular patterns (DAMPs) and preservation of tumor-associated antigens (TAAs). These molecular signals recruit and activate antigen-presenting cells, promote tumor-specific T-cell priming, and may induce systemic anti-tumor immunity. This mechanism suggests that PEF could act synergistically with immunotherapeutic agents, potentially overcoming immune resistance mechanisms observed in “cold” or immune checkpoint inhibitor-refractory tumors [6]. In murine tumor models, PEF has been shown to promote both local tumor regression and the regression of distant, untreated lesions via an abscopal effect, suggesting a potential role in inducing long-lasting systemic immunity [7,8]. The INCITE ES study demonstrated initial safety and feasibility of PEF in adult patients and suggested similar engagement of host innate and adaptive immune responses [9]. In addition, emerging six-month data from the AFFINITY trial showed robust activation of both innate and humoral immune responses, persisting across multiple post-treatment intervals [10,11]. PEF ablation may also be particularly well-suited for oligometastatic stage IV lung cancer patients, in whom there is increasing evidence that local treatment of primary tumor sites may provide survival benefit over systemic therapy alone [12].

Given its dual mechanism of tumor ablation and potential immune activation, PEF therapy occupies a unique intersection of interventional oncology and cancer immunotherapy. Further evaluation in real-world clinical settings is critical to defining optimal patient selection, treatment timing, and integration with immune-based systemic therapies. Herein, we present our early experience with PEF ablation in a community hospital setting, reporting outcomes from the first 32 consecutive patients with primary lung cancer or oligometastatic disease involving the lung who underwent PEF ablation after progression on front line therapy, and also in patients who had no other options for treatment for their early-stage disease.

## 2. Materials and Methods

This retrospective study reviewed medical records of all patients who underwent PEF ablation at our institution from 7 May 2024 to 1 May 2025. The first 32 consecutive patients were included. This number was chosen for two reasons: (1) these were the only patients who had reached six-month follow-up by the time of manuscript submission, and (2) Eisenhower Health had recently joined the PROPEL registry, and it was predetermined that patients included in this case series could not simultaneously be included in the registry. Demographic information, cancer type and stage, molecular studies (when available), treatment history and response, follow-up imaging at roughly 3 and 6 months from ablation, and outcomes for patients treated using PEF ablation therapy were collected. Patients were eligible if they had progressive disease despite front-line systemic therapy or if they had early-stage disease with no other options (not surgical or radiation candidates, or patient preference). Patients underwent PEF ablation with the intention to control local or oligometastatic thoracic disease.

### 2.1. Device Use and Early Off-Label Application

Although the Aliya PEF system (Galvanize Therapeutics, Inc, Redwood City, CA, USA) itself was FDA 510(k) cleared at the time of treatment of all patients, the first three patients in this series underwent bronchoscopic PEF ablation prior to FDA 510(k) clearance of the Inumi Flex endoscopic needle (Galvanize Therapeutics, Inc., Redwood City, CA, USA) (cleared on 28 May 2024). As a result, energy delivery for these early cases was performed using commercially available needles with similarly non-insulated tips as the Inumi Flex needle, but not cleared for PEF application. Specifically, Patients 1 and 3 were treated using a 21 G Periview Flex needle (Olympus, Tokyo, Japan), and Patient 2 was treated using a 19 G ViziShot 2 needle (Olympus, Tokyo, Japan). All three patients were not candidates for percutaneous PEF delivery, which was the only FDA-cleared route at that time. Each patient had progressive disease despite multiple prior lines of chemotherapy and immunotherapy, and all were appropriately counseled and consented regarding the off-label nature of bronchoscopic energy delivery.

All patients underwent robotic-assisted bronchoscopy using the Ion robotic platform (Intuitive Surgical, Sunnyvale, CA, USA) with fluoroscopy and EBUS or integrated CT imaging (Cios Spin, Siemens Medical Solutions, Malvern, PA, USA). After navigation to the target lesion using pre-procedural planning and real-time bronchoscopic guidance, imaging was acquired to confirm accurate catheter positioning within the lesion by CBCT. A diagnostic sample was obtained with a flexible needle or transbronchial biopsy, reviewed by rapid-onsite evaluation (ROSE), with confirmation of malignancy. In all cases, except the first three cases as previously described, the FDA-cleared Inumi needle was then deployed through the working channel, with additional CBCT spins obtained to confirm needle position at the distal margin of the lesion in that vector. The Aliya PEF System was used to deliver a standardized, preset PEF dose consisting of 3 kV and 100 cardiac-gated energy packets, lasting ~5 min. The needle was adjusted to create overlapping ablation zones as it was withdrawn by 1 cm at a time, as measured by fluoroscopic image. For larger lesions and when technically feasible, multiple vectors were utilized with a goal of maximal lesion ablation while avoiding critical adjacent structures.

### 2.2. Radiologic Response Assessment

Tumor response was assessed according to RECIST 1.1 using the Sum of Longest Diameters (SLD) to categorize patient response to treatment as either progressive disease (PD), stabilization of disease (SD), partial response (PR), or complete response (CR):

- Complete Response (CR): Disappearance of all target lesions. Any pathological lymph nodes must decrease to <10 mm in short axis.
- Partial Response (PR): At least a 30% decrease in the sum of longest diameters (SLD) of target lesions, taking as reference the baseline SLD.
- Progressive Disease (PD): At least a 20% increase in SLD, taking as reference the smallest SLD recorded (nadir) AND an absolute increase of at least 5 mm. Progression can also be defined by the appearance of new lesions.
- Stable Disease (SD): Neither sufficient shrinkage to qualify for PR nor sufficient increase to qualify for PD [13,14].

Radiographic evidence of potential abscopal response was determined through evaluation of non-target lesion(s) according to RECIST 1.1 SLD. No serum biomarkers or immune profiling were conducted in this specific cohort to biologically substantiate potential abscopal responses.

Volumetric measurements were also collected whenever possible using Ion^TM^ PlanPoint software version 4.0.0 (Intuitive Surgical, Sunnyvale, CA, USA) and Riverain ClearRead^TM^ software version 5.8.4 (Riverain Technologies, Miamisburg, OH, USA). Because this technology only ablates/treats a single lesion at a time, and at times these lesions may be less than or approximately one centimeter, percent volumetric change thresholds corresponding to standard RECIST 1.1 categories were applied as “volumetric RECIST”, herein termed “vRECIST”. For patients in whom multiple lesions were treated, SLD was calculated using the longest diameter from each lesion and volumetric measurements involved the sum of the volumes of treated lesions. SLD and volumetric measurements were calculated with assistance of software from Ion Plan Manager and Riverain technology wherever possible.

### 2.3. Follow-Up and Statistical Analysis

Data was collected at two follow-up intervals: approximately 3 months and 6 months post-treatment in all patients. Descriptive statistics, including means, medians, standard deviations, and proportions, were used. As a retrospective analysis of a sequential case series without control for differences in systemic therapies or between patients and given the heterogenous cohort, no inferential statistics were performed, and the outcomes data remains hypothesis-generating rather than efficacy evidence.

## 3. Results

### 3.1. Initial Follow-Up (~3 Months)

Thirty-two patients, 19 men and 13 women, all with progressive disease despite therapy, were treated with PEF ablation during the study period. Patient characteristics are summarized in Table 1 below, with further individual data available in Supplementary Tables S1 and S2. Thirty of these patients had subsequent collection of initial follow-up data, while two unfortunately died prior to follow-up. Neither of the deceased patients suffered complications linked to the procedure. Median (IQR) follow-up time was 88.5 (74–95) days. According to standard RECIST 1.1 criteria, at follow-up, 6.3% (2/32) of patients showed complete response, 15.6% (5/32) showed partial response, and 59.4% (19/32) showed stable disease. Only 12.5% (4/32) of patients showed progressive disease at initial follow-up, with an additional 6.3% (2/32) of patients having passed prior to follow-up. Thus, 7 of 32 patients had complete or partial response to treatment, while 26 of 32 patients had stabilization of disease, a partial response, or complete response to treatment, yielding an ORR of 21.88% and a disease control rate/clinical benefit of 81.25%. Of note, 27.6% (8/29) of the patients with Stage III or IV cancer had radiographic evidence of decreased size in non-treated lesions, suggestive of a possible abscopal response.

### 3.2. Second Follow-Up (~6 Months)

Subsequent follow-up data was collected for 24 of the initial 32 patients, as a further 6 died prior to the second follow-up period. Median (IQR) follow-up time was 180.5 (158–207) days. On follow-up, 6.3% (2/32) of patients showed complete response, 12.5% (4/32) showed partial response, and 43.8% (14/32) showed stable disease. Only 12.5% (4/32) of patients showed progressive disease at follow-up, with an additional 25.0% (8/32) deceased since initial treatment. Thus, 20 of the initial 32 patients lived to 6-month follow-up and had stabilization of disease, a partial response, or complete response to treatment, yielding an ORR of 18.75% and a disease control rate/clinical benefit of 62.5%. This data is summarized numerically in Table 2, and provided on an individual basis in Table 3 and Table 4. For the 24 evaluable patients at 6 months, this DCR becomes 83.3% (20/24) with stabilization of disease or better.

Of note, 5 of the 24 patients in whom second follow-up data was collected did receive a second treatment prior to the second follow-up period, 3 demonstrated stable disease at initial follow-up, 1 demonstrated partial response, and 1 demonstrated progressive disease. Excluding these patients from the second analysis yields 7.4% (2/27) of patients with complete response, 11.1% (3/27) with partial response, and 37.0% (10/27) with stable disease. Only 14.8% (4/27) of patients showed progressive disease at follow-up, with an additional 29.6% (8/27) deceased since initial treatment. Thus, 15 of the 27 patients lived to 6-month follow-up and had stabilization of disease, a partial response, or complete response to treatment, suggesting a clinical benefit of 55.6% even after removing the re-treated patients. Despite this promising data, it is important to note that long-term oncologic outcomes and comparative effectiveness require prospective, controlled investigation.

### 3.3. Volumetric Reclassification at 3 and 6 Months

Many post-PEF lesions classified as stable disease showed a negative trend, and % change in volume has been proposed as a more sensitive metric than Sum of Longest Dimensions [9]. This was borne out in our volumetric measurement data: at 3 months, 7 patients with stable disease under RECIST 1.1 would be reclassified as having partial response by volumetric data. The sensitivity cut both ways, with two patients with stable disease being reclassified as having progressive disease. For the 6-month follow-up group, volumetric measurement data in comparison to RECIST 1.1 was similar to that in the 3-month follow-up group, with six patients with stable disease being reclassified as having partial response, and three being reclassified as having progressive disease.

In comparing 3-month to 6-month volumetric follow-up data, 66.7% (16/24) patients remained in the same response category according to RECIST 1.1. Of the eight who changed, three had improvement to partial response (two from stable disease, one from progressive disease), one had improvement to stable disease (from progressive disease), one had worsening to stable disease from partial response, and three had worsening to progressive disease (two from stable disease, one from partial response). Of the five patients who were re-treated prior to second follow-up, four remained in the same RECIST 1.1 category at 6 months compared to at 3 months, all at stable disease, while one improved from stable disease to partial response.

**Figure 1:**
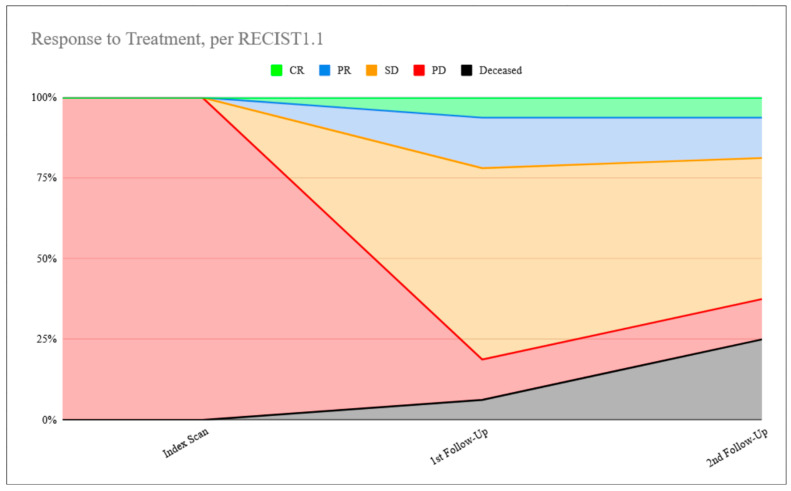
Response to treatment, per RECIST1.1.

**Figure 2:**
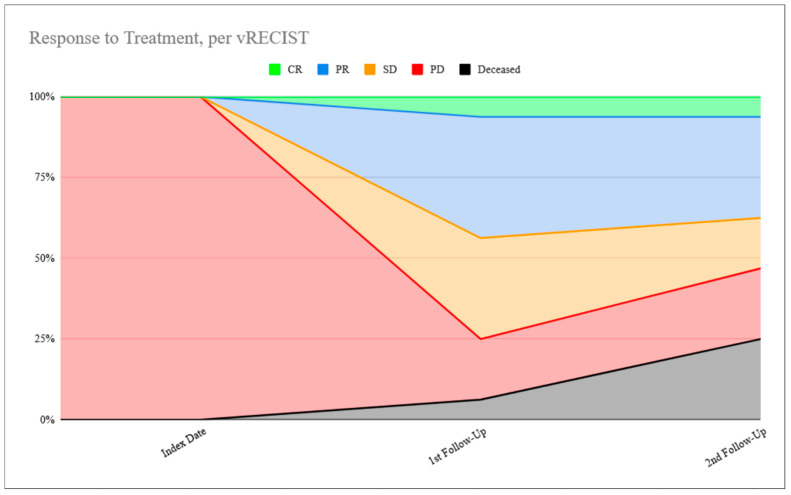
Response to treatment, per vRECIST.

This same assessment of the volumetric data, comparing 3-month to 6-month follow-up data, yields 58.3% (14/24) patients in the same response category per vRECIST. Of the 10 who changed, five had improvement to partial response (three from stable disease, two from progressive disease), one had worsening to stable disease (from partial response), and four had worsening to progressive disease (two from stable disease, two from partial response). Figure 1 and Figure 2 demonstrate this response to treatment over time according to both RECIST1.1 and vRECIST. Table 2 summarizes the data side-by-side numerically.

## 4. Complications

Of 32 patients, 28 tolerated the procedure entirely without complications. One developed mild pneumomediastinum post-procedure, which self-resolved under observation. One developed pleuritic pain post-procedure, which was resolved with conservative management. One developed periprocedural bleeding, grade 2, which was controlled during the procedure without further complication and without requiring transfusion. Only one patient had a serious complication as a possible result of PEF ablation. This complication occurred in a patient with rapidly progressive stage IIIB squamous cell carcinoma of the right lower lobe of the lung with poor prognosis. The tumor had penetrated the posterolateral wall of the bronchus intermedius, resulting in complete obstruction of the right middle and lower lobe bronchi. Restoration of airway patency required extensive endobronchial tumor debulking with cryoprobe and cryotherapy prior to PEF ablation with the Aliya device. Three weeks later, the patient presented with hemoptysis, and bronchoscopy on 27 June 2024 revealed a fistulous tract from the bronchus intermedius into the cavitary right lower lobe mass, accompanied by pneumothorax and a persistent air leak consistent with a bronchopleural fistula (BPF). Cultures from the index procedure on 6 June 2024 grew Aspergillus terreus. Although the Fungitell assay was initially negative at the time of readmission, it became positive (>500 pg/mL) on 1 July 2024. The patient transitioned to hospice in September 2024, with no further follow-up. Given the presence of a rapidly progressing cavitary tumor, prior cryotherapy and cryobiopsies, and evidence of invasive aspergillus infection, it remains unclear whether the BPF resulted from PEF ablation itself or from one or more of these concurrent processes.

## 5. Discussion

With our early adoption of PEF therapy, the purpose of this study was to report a community hospital’s experience with the Aliya system to help broaden understanding of those who might benefit from this technology. Given that our patient population selected to receive PEF treatment was composed primarily of patients with progressive disease despite systemic therapy, our initial results are very promising. In addition, excellent local control was demonstrated in the few patients included that had early-stage disease who were not on systemic therapy; 81.25% of patients had improvement to at least stabilization of disease per RECIST 1.1 at 3-month follow-up, with this benefit level demonstrated in 62.5% of the cohort at 6-month follow-up. While five patients did receive a second treatment between the follow-up periods, even excluding these patients from the 6-month follow-up analysis results in a promising 55.6% of patients with stabilization of disease, partial response, or complete response. Given the excellent safety profile and low rate of complications with PEF ablation, and the lack of barriers to re-treatment compared to radiofrequency or other thermal ablation modalities, we recommend further exploration of re-treatment, particularly in a patient population such as ours, with previously progressive disease despite systemic therapy. That said, despite this promising data, it is important to note that long-term oncologic outcomes and comparative effectiveness require prospective, controlled investigation.

Overall, 27.6% (8 of 29) of the patients with Stage III or IV cancer demonstrated radiographic findings consistent with possible abscopal responses at 3 months with decreased size in non-treated lesions, though it is important to note that no serum biomarkers or immune profiling was conducted in this specific cohort to biologically substantiate potential abscopal responses. Nonetheless, these findings align with PEF’s immunogenic potential, as supported by both preclinical literature and emerging clinical trial data. Our findings are consistent with a previously published study by William Moore and colleagues in a propensity-matched study of patients with refractory stage IV non-small cell lung cancer [15].

Analysis of volumetric data also proves interesting, as many post-PEF lesions classified as stable disease do show a strong negative or positive trend. Percentage change in volume has been proposed as a more sensitive metric than SLD, allowing for a more accurate representation of tumor size and changes over time [16]. Volumetric measurement has also demonstrated better inter-reader agreement [17,18], and may be preferred for smaller nodules and much larger nodules, where a relatively small variation in diameter can cause a large variation in volume. Volumetric measurement calculations on our patients did result in re-classification of many of these patients—36.7% (11/30) at 3-month follow-up and 37.5% (9/24) at 6 months. It is important to note that RECIST 1.1 has significant limitations in ablative contexts. Post-ablative imaging has to contend with the fact that tumor size does not necessarily directly correlate with tissue viability, post-ablation inflammation and necrosis can lead to apparent enlargement, RECIST assumes tumors are measurable in a reproducible linear fashion when this may not be the case and many tumors have irregular geometry, and many image guided focal therapies can leave scars similar in size to treated lesions [19,20,21,22]. Nonetheless, despite research into functional imaging and other forms of assessment, there remains a significant role for post-ablation imaging monitoring, with ablation zones expected to be smaller than baseline tumors by 6 months, with more profound early shrinkage seen in cryoablation compared to radiofrequency and microwave ablation [23,24]. Ion and Riverain software have not been specifically externally validated for PEF-induced necrosis and inflammation, but although there is limited validation data, there is significant potential for volumetric analysis to help address some of the larger deficiencies related to imaging post-ablation, particularly with respect to irregularly shaped lesions. These findings form the basis for our recommendation for the integration of volumetric response criteria into future PEF trials.

There are several limitations to this analysis. As a retrospective analysis of a sequential case series without control for differences in systemic therapies or between patients, there is limited ability to determine statistical significance. The patient population was very heterogeneous, comprising 13 different cancers with varied molecular profiles. There was no control of systemic therapies before and after PEF, and five patients underwent a second PEF ablation prior to the 6-month follow-up period. Despite these constraints, the consistency of disease control across diverse tumor types supports the broad potential applicability of PEF. Only one patient experienced a serious complication—the bronchopleural fistula described above. Following this event, expert consensus and emerging guidance recommended avoiding PEF treatment in tumors that constitute part of the airway wall. Our patient selection criteria were subsequently refined to exclude lesions with airway invasion to the point of breakdown of normal stromal barrier function; in patients with mixed endoluminal and extraluminal disease, only extraluminal disease was targeted. After implementing these changes, no additional serious complications occurred—either within the remainder of this cohort or among the subsequent 44 patients treated at our center and entered into the PROPEL registry.

Overall, PEF ablation presents an attractive option within the clinical field of ablative cancer treatment. Local thermal ablation therapies such as microwave, radiofrequency, and cryoablation carry meaningful risks of multiple complications, including hemorrhage, damage to nearby critical structures, radiation pneumonitis, and death [24,25,26,27]. PEF ablation has a significantly lower risk of complications in close proximity to critical structures, and to date the safety of bronchoscopic PEF has been very favorable, as described in multiple small studies [4,28,29,30]. Despite the limitations discussed above, our study adds to this body of literature, demonstrating the real-world safety and feasibility of PEF ablation across a wide variety of cancer-types, including local and especially advanced disease, given the potential immunogenic enhancement. However, unanswered questions regarding PEF ablation remain, necessitating further research to identify the cancers and patients most likely to benefit from therapy, the optimal timing of therapy, and the role of immunotherapy in conjunction with ablation.

## 6. Conclusions

In this retrospective study at our community hospital, pulsed electric field ablation was safe and demonstrated meaningful clinical benefit in patients with early-stage lung cancer with no other options and in patients with advanced lung cancer or with oligometastatic thoracic disease progressing despite front-line therapy. Good rates of disease control, possible evidence of systemic immune effects, and favorable tolerability as well as safety profiles highlight the promise of PEF ablation both for local control and to enhance immunologically active therapy. Prospective controlled studies are warranted to better define optimal patient selection, treatment sequencing, and integration with systemic immunotherapy.

## Figures and Tables

**Table 1:** Cohort characteristics.

Median (IQR) Age	76.5 (68–81.5) years old
Sex	19 Male 13 Female
Cancer Type	9 Adenocarcinoma, Lung 2 Squamous Cell, Lung 1 Large Cell Neuroendocrine 2 Carcinoid 3 Squamous Cell, Head and Neck 1 Squamous Cell, Vagina 1 Leiomyosarcoma, Vaginal 1 Breast 3 Colorectal 6 Renal Cell 1 Urothelial 1 B-Cell Lymphoma 1 Melanoma
Cancer Stage	Stage I: 2 patients Stage II: 1 patient Stage III: 3 patients Stage IV: 26 patients
Radiographic Evidence of Possible Abscopal Response	27.6% (8/29 patients w/Stage III/IV cancer)
Procedural Complications	4/32 patients

**Table 2:** Tumor response at first and second follow-up, according to RECIST 1.1 and vRECIST.

	1st Follow-Up (~3 Months)		2nd Follow-Up (~6 Months)	
	RECIST 1.1	vRECIST	RECIST 1.1	vRECIST
Deceased	6.3% (2/32)	6.3% (2/32)	25.0% (8/32)	25.0% (8/32)
PD	12.5% (4/32)	18.8% (6/32)	12.5% (4/32)	21.9% (7/32)
SD	59.4% (19/32)	31.3% (10/32)	43.8% (14/32)	15.6% (5/32)
PR	15.6% (5/32)	37.5% (12/32)	12.5% (4/32)	31.3% (10/32)
CR	6.3% (2/32)	6.3% (2/32)	6.3% (2/32)	6.3% (2/32)

**Table 3:** Patient-by-patient response data at initial follow-up. Blacked out cells represent patients who died prior to follow-up.

Case No.	Cancer Type	# of Ablations	1st Follow-Up (Days)	Change in SOD	RECIST 1.1	Change in Volume	vRECIST
1	Squamous Cell Lung	6	74	−5.71%	SD	−22.39%	SD
2	Adenocarcinoma Lung	6, 3	97	−21.05%	SD	−48.68%	PR
3	Squamous Vaginal	4, 4	82	−23.53%	SD	−69.88%	PR
4	Squamous Cell Lung	11	28	−43.62%	PR	−63.97%	PR
5	Breast Cancer, Triple Negative	10					
6	B-Cell Lymphoma	3, 2	77	−100.00%	CR	−100.00%	CR
7	Squamous Head and Neck	12	174	−2.08%	SD	−16.87%	SD
8	Renal Cell	4	94	0.00%	SD	−57.14%	PR
9	Adenocarcinoma Lung	8	102	−11.54%	SD	−20.72%	SD
10	Adenocarcinoma Lung	11	93	6.90%	SD	4.65%	SD
11	Leiomyosarcoma, vaginal source	10, 12	81	4.69%	SD	11.60%	SD
12	Colorectal	2	95	−100.00%	CR	−100.00%	CR
13	Adenocarcinoma Lung	6	60	−10.00%	SD	−19.75%	SD
14	Adenocarcinoma Lung	7	96	65.22%	PD	295.92%	PD
15	Renal Cell, Papillary type	4, 6	89	0.00%	SD	4.26%	SD
16	Melanoma	3	53	30.00%	PD	115.82%	PD
17	Renal Cell, Clear Cell	9	73	−32.14%	PR	−59.83%	PR
18	Squamous Head and Neck (HPV+)	4, 1, 5, 4	123	3.03%	SD	20.46%	PD
19	Adenocarcinoma Lung	7, 4, 5					
20	Squamous Head and Neck (HPV+)	10	61	−58.00%	PR	−95.55%	PR
21	Colon	10	96	22.22%	SD	13.35%	SD
22	Urothelial Carcinoma	4, 7	89	47.06%	PD	166.65%	PD
23	Large Cell Neuroendocrine	6	95	33.33%	PD	16.32%	SD
24	Carcinoid	4, 4	96	−36.67%	PR	−79.77%	PR
25	Carcinoid	12	91	−23.53%	SD	−58.86%	PR
26	Adenocarcinoma Lung (acinar and papillary pattern)	10	23	3.23%	SD	−50.54%	PR
27	Renal Cell	16	95	−29.85%	SD	−65.64%	PR
28	Adenocarcinoma Lung	8	55	−8.51%	SD	−1.46%	SD
29	Renal Cell	3	83	−44.44%	PR	−80.28%	PR
30	Colorectal adenocarcinoma	4, 11	87	−5.41%	SD	44.98%	PD
31	Adenocarcinoma Lung (mucinous features)	7	79	16.67%	SD	23.20%	PD
32	Renal Cell	4	88	−19.05%	SD	−52.90%	PR

**Table 4:** Patient-by-patient response data at second follow-up, with overall complications. Blacked out cells represent patients who died prior to follow-up.

Case No.	Cancer Type	2nd Follow-Up (Days)	Change in SOD	RECIST 1.1	Change in Volume	vRECIST	Complications
1	Squamous Cell Lung						None
2	Adenocarcinoma Lung						None
3	Squamous Vaginal	82	23.53%	PD	72.60%	PD	None
4	Squamous Cell Lung						Airway perf w/BPF
5	Breast Cancer, Triple Negative						None
6	B-Cell Lymphoma	211	−100.00%	CR	−100.00%	CR	None
7	Squamous Head and Neck	292	−27.08%	SD	−57.14%	PR	Pneumomediastinum
8	Renal Cell	216	−12.50%	SD	−66.25%	PR	None
9	Adenocarcinoma Lung	189	−11.54%	SD	−28.36%	SD	Pleuritic pain
10	Adenocarcinoma Lung	179	10.34%	SD	−18.39%	SD	None
11	Leiomyosarcoma, vaginal source	173	12.50%	SD	40.62%	PD	None
12	Colorectal	191	−100.00%	CR	−100.00%	CR	None
13	Adenocarcinoma Lung	182	−15.00%	SD	−37.52%	PR	Bleeding, Grade 2
14	Adenocarcinoma Lung	268	−39.13%	PR	−71.47%	PR	None
15	Renal Cell, Papillary type	157	1.89%	SD	33.20%	PD	None
16	Melanoma						None
17	Renal Cell, Clear Cell	203	−25.00%	SD	−45.59%	PR	None
18	Squamous Head and Neck (HPV+)	175	46.97%	PD	261.97%	PD	None
19	Adenocarcinoma Lung						None
20	Squamous Head and Neck (HPV+)	159	N/A	PD	N/A	PD	None
21	Colon	174	16.67%	SD	3.34%	SD	None
22	Urothelial Carcinoma	244	91.18%	PD	746.08%	PD	None
23	Large Cell Neuroendocrine	187	−19.05%	SD	−54.49%	PR	None
24	Carcinoid	193	−50.00%	PR	−86.19%	PR	None
25	Carcinoid	220	−38.24%	PR	−82.96%	PR	None
26	Adenocarcinoma Lung (acinar and papillary pattern)						None
27	Renal Cell	154	−7.46%	SD	−28.32%	SD	None
28	Adenocarcinoma Lung	136	−29.79%	SD	−4.26%	SD	None
29	Renal Cell						None
30	Colorectal adenocarcinoma	120	8.11%	SD	112.93%	PD	None
31	Adenocarcinoma Lung (mucinous features)	152	−33.33%	PR	−80.33%	PR	None
32	Renal Cell	179	−23.81%	SD	−48.05%	PR	None

## Data Availability

Research data available in Supplemental Materials.

## Supplementary Materials

The following supporting information can be downloaded at https://www.mdpi.com/article/10.3390/cancers18091459/s1, Table S1: Supplemental Data Table—Individual; Table S2: Supplemental Data Table—Markers.

## Abbreviations

The following abbreviations are used in this manuscript:

PEF	Pulsed Electric Field
RECIST	Response Evaluation Criteria in Solid Tumors
CR	Complete Response
PR	Partial Response
SD	Stable Disease
PD	Progressive Disease
