# Supplementary material for: Pulsed Electric Field Ablation for Advanced Lung and Oligometastatic Disease: A Retrospective Study of 32 Consecutive Patients in a Community Hospital Setting
Source: Cancers (Basel). 2026 May 1;18(9):1459. doi: 10.3390/cancers18091459 (PMC13162631; doi:10.3390/cancers18091459)
Supplement: Supplementary file 1 [file cancers-18-01459-s001.zip › Table S1 - Supplemental Data Table - Individual.pdf]

Supplemental Data Table - Individual

Case	Age	Sex	Cancer	# of Ablations	Pre-PEF Size (mm)			Pre-PEF SOD (mm)	Pre-PEF Volume (mm3)	1st F/U Period (days)	1st F/U Post-PEF Size (mm)			1st F/U Post-PEF SOD (mm)	1st F/U Post-PEF Volume (mm3)	1st F/U SLD % Change	1st F/U Response per RECIST 1.1	1st F/U Volume % Change	1st F/U Response per vRECIST	2nd F/U Period (days)
1	72	F	Squamous Cell Lung	6	70	44	64	70	103,212	74	66	38	61	66	80,104	-5.71%	SD	-22.39%	SD	[redacted]
2a	71	F	Adenocarcinoma Lung	6	18	11	14	18	1,451	97	13	7	13	13	619	-27.78%	-	-57.32%	-	[redacted]
2b	-	-	-	3	15	17	20	20	2,670	-	14	12	17	17	1,495	-15.00%	-	-44.00%	-	[redacted]
Total	-	-	-	-	-	-	-	38	4,121	-	-	-	-	30	2,115	-21.05%	SD	-48.68%	PR	[redacted]
3a	77	F	Squamous Vaginal	4	19	22	18	22	3,940	82	17	8	16	17	1,139	-22.73%	-	-71.08%	-	82
3b	-	-	-	4	11	12	12	12	829	-	9	7	9	9	297	-25.00%	-	-64.20%	-	-
Total	-	-	-	-	-	-	-	34	4,769	-	-	-	-	26	1,436	-23.53%	SD	-69.88%	PR	-
4	69	M	Squamous Cell Lung	11	53	62	94	94	161,731	28	42	50	53	53	58,277	-43.62%	PR	-63.97%	PR	[redacted]
5	86	F	Breast Cancer, triple negative	10	unable to assess			0	N/A	[redacted]										
6a	68	M	B-Cell Lymphoma	3	17	21	14	21	2,547	77	0	0	0	0	0	-100.00%	-	-100.00%	-	211
6b	-	-	-	2	14	14	12	14	1,058	-	0	0	0	0	0	-100.00%	-	-100.00%	-	-
Total	-	-	-	-	-	-	-	35	3,605	-	-	-	-	0	0	-100.00%	CR	-100.00%	CR	-
7	81	M	Squamous Head and Neck	12	48	42	35	48	36,945	174	39	32	47	47	30,712	-2.08%	SD	-16.87%	SD	292
8	88	F	Renal Cell	4	16	14	15	16	1,759	94	16	10	9	16	754	0.00%	SD	-57.14%	PR	216
9	76	F	Adenocarcinoma Lung	8	21	26	23	26	4,859	102	20	23	18	23	3,852	-11.54%	SD	-20.72%	SD	189
10	67	F	Adenocarcinoma Lung	11	29	27	29	29	10,534	93	31	30	26	31	11,024	6.90%	SD	4.65%	SD	179
11a	46	M	Leiomyosarcoma, vaginal source	10	33	35	33	35	18,827	81	36	35	33	36	21,074	2.86%	-	11.93%	-	173
11b	-	-	-	12	29	29	28	29	11,363	-	29	31	29	31	12,617	6.90%	-	11.04%	-	-
Total	-	-	-	-	-	-	-	64	30,190	-	-	-	-	67	33,691	4.69%	SD	11.60%	SD	-
12	82	F	Rectal	2	8	9	8	9	341	95	0	0	0	0	0	-100.00%	CR	-100.00%	CR	191
13	79	M	Adenocarcinoma Lung	6	17	18	20	20	3,297	60	16	18	17	18	2,646	-10.00%	SD	-19.75%	SD	182
14	77	M	Adenocarcinoma Lung	7	23	14	18	23	2,625	96	38	25	24	38	10,393	65.22%	PD	295.92%	PD	268
15a	71	M	Renal Cell, Papillary	4	26	18	27	27	6,616	89	25	18	27	27	6,362	0.00%	-	-3.85%	-	157
15b	-	-	-	6	26	16	17	26	3,703	-	26	19	17	26	4,397	0.00%	-	18.75%	-	-
Total	-	-	-	-	-	-	-	53	10,319	-	-	-	-	53	10,759	0.00%	SD	4.26%	SD	-
16	85	M	Melanoma	3	10	9	9	10	474	53	13	11	11	13	1,023	30.00%	PD	115.82%	PD	[redacted]
17	67	M	Renal Cell, Clear Cell	9	20	28	15	28	5,073	73	14	19	12	19	2,038	-32.14%	PR	-59.83%	PR	203
18a	68	M	Squamous Head and Neck, HPV+	4	14	12	13	14	1,092	123	14	15	12	15	1,083	7.14%	-	-0.82%	-	175
18b	-	-	-	1	17	12	17	17	1,952	-	23	20	17	23	4,025	35.29%	-	106.20%	-	-
18c	-	-	-	5	14	15	17	17	1,863	-	15	15	13	15	1,489	-11.76%	-	-20.08%	-	-
18d	-	-	-	4	14	13	18	18	1,701	-	14	13	15	15	1,363	-16.67%	-	-19.87%	-	-
Total	-	-	-	-	-	-	-	66	6,608	-	-	-	-	68	7,960	3.03%	SD	20.46%	PD	-
19a	68	M	Adenocarcinoma Lung	7	20	18	32	32	6,032	[redacted]										
19b	-	-	-	4	15	12	14	15	1,319	[redacted]										
19c	-	-	-	5	18	18	15	18	2,545	[redacted]										
Total	-	-	-	-	-	-	-	65	9,896	[redacted]										
20	86	M	Squamous Head and Neck, HPV+	10	50	47	41	50	50,449	61	17	21	12	21	2,243	-58.00%	PR	-95.55%	PR	159
21	81	M	Colon	10	17	18	17	18	2,757	96	15	22	17	22	3,125	22.22%	SD	13.35%	SD	174
22a	87	F	Urothelial Carcinoma	4	16	13	16	16	1,743	89	31	21	27	31	9,203	93.75%	-	428.16%	-	244
22b	-	-	-	7	17	17	18	18	2,724	-	17	16	19	19	2,706	5.56%	-	-0.65%	-	-
Total	-	-	-	-	-	-	-	34	4,466	-	-	-	-	50	11,909	47.06%	PD	166.65%	PD	-
23	80	F	Large Cell Neuroendocrine	6	17	19	21	21	3,744	95	28	19	17	28	4,355	33.33%	PD	-79.38%	SD	187
24a	49	M	Carcinoid	4	16	12	13	16	1,062	96	10	7	6	10	219	-37.50%	-	-80.15%	-	193
24b	-	-	-	4	14	13	13	14	1,088	-	9	7	7	9	216	-35.71%	-	-79.77%	-	-
Total	-	-	-	-	-	-	-	30	2,150	-	-	-	-	19	435	-36.67%	PR	3.35%	PR	-
25	80	M	Carcinoid	12	20	29	34	34	10,325	91	24	13	26	26	4,247	-23.53%	SD	-58.86%	PR	220
26	86	F	Adenocarcinoma Lung, acinar and	10	31	24	31	31	12,843	23	32	16	18	32	6,352	3.23%	SD	-50.54%	PR	[redacted]
27	75	M	Renal Cell	16	64	61	67	67	136,957	95	47	43	45	47	47,065	-29.85%	SD	-65.64%	PR	154
28	75	M	Adenocarcinoma Lung	8	22	33	47	47	17,866	55	23	34	43	43	17,607	-8.51%	SD	-1.45%	SD	136
29	83	F	Renal Cell	3	15	14	18	18	2,490	83	9	7	10	10	491	-44.44%	PR	-80.28%	PR	[redacted]
30a	66	M	Colorectal Adenocarcinoma	4	17	16	14	17	1,284	87	18	18	18	18	2,914	5.88%	-	126.95%	-	120
30b	-	-	-	11	57	28	27	57	17,010	-	32	32	52	52	23,608	-8.77%	-	38.79%	-	-
Total	-	-	-	-	-	-	-	74	18,294	-	-	-	-	70	26,522	-5.41%	SD	44.98%	PD	-
31	32	F	Adenocarcinoma Lung, mucinous f	7	12	11	12	12	905	79	12	13	14	14	1,115	16.67%	SD	23.20%	PD	152
32	81	M	Renal Cell	4	21	11	20	21	2,419	88	16	8	17	17	1,139	-19.05%	SD	-52.90%	PR	179

Case	2nd F/U Post-PEF Size (mm)			2nd F/U Post-PEF SOD (mm)	2nd F/U Post-PEF Volume (mm3)	2nd F/U SLD % Change	2nd F/U Response per RECIST 1.1	2nd F/U Volume % Change	2nd F/U Response per vRECIST	Abscopal Response	Complications	Additional Comments
1	[redacted]									No	No	Deceased prior to second follow-up.
2a										-	-	-
2b										-	-	-
Total										No	No	Deceased prior to second follow-up. 2 treated lesions.
3a	16	14	14	16	1,642	-27.27%	-	-58.32%	-	-	-	-
3b	22	22	26	26	6,589	116.67%	-	694.44%	-	-	-	-
Total	-	-	-	42	8,231	23.53%	PD	72.60%	PD	Yes	No	2 treated lesions.
4	[redacted]									No	Airway perf with BPF	Deceased prior to second follow-up.
5										N/A	No	Deceased prior to initial follow-up. Not able to measure size of tumor as it is large with complete right hemithorax
6a	0	0	0	0	0	-100.00%	-	-100.00%	-	-	-	-
6b	0	0	0	0	0	-100.00%	-	-100.00%	-	-	-	-
Total	-	-	-	0	0	-100.00%	CR	-100.00%	CR	Yes	No	2 treated lesions.
7	35	32	27	35	15,834	-27.08%	SD	-57.14%	PR	No	Pneumomediastinum	None
8	14	9	9	14	594	-12.50%	SD	-66.25%	PR	Yes	No	None
9	23	19	21	23	3,481	-11.54%	SD	-28.36%	SD	N/A	Pleuritic pain	None
10	32	27	27	32	8,597	10.34%	SD	-18.39%	SD	Yes	No	None
11a	39	38	36	39	25,550	11.43%	-	35.71%	-	-	-	-
11b	33	33	31	33	16,903	13.79%	-	48.75%	-	-	-	-
Total	-	-	-	72	42,453	12.50%	SD	40.62%	PD	No	No	2 treated lesions.
12	0	0	0	0	0	-100.00%	CR	-100.00%	CR	N/A	No	None
13	13	17	15	17	2,060	-15.00%	SD	-37.52%	PR	No	Bleeding (Grade 2)	None
14	11	9	14	14	749	-39.13%	PR	-71.47%	PR	No	No	None
15a	18	25	29	29	6,833	7.41%	-	3.28%	-	-	-	-
15b	22	25	24	25	6,912	-3.85%	-	86.65%	-	-	-	-
Total	-	-	-	54	13,745	1.89%	SD	33.20%	PD	No	No	2 treated lesions. Re-treated before 2nd f/u.
16	[redacted]									No	No	Deceased prior to second follow-up.
17	17	21	16	21	2,760	-25.00%	SD	-45.59%	PR	Yes	No	None
18a	17	18	16	18	2,302	28.57%	-	110.81%	-	-	-	-
18b	28	22	23	28	7,090	64.71%	-	263.22%	-	-	-	-
18c	17	17	19	19	2,607	11.76%	-	39.94%	-	-	-	-
18d	32	30	26	32	11,920	77.78%	-	600.76%	-	-	-	-
Total	-	-	-	97	23,919	46.97%	PD	261.97%	PD	No	No	4 treated lesions.
19a	[redacted]									-	-	-
19b										-	-	-
19c										-	-	-
Total										N/A	No	Deceased prior to initial follow-up.
20	N/A	N/A	N/A	N/A	N/A	N/A	PD (comment)	N/A	PD (comment)	Yes	No	2nd F/U without volume numbers due to RUL collapse, but metabolic progression + nodal enlargement on PET.
21	16	16	21	21	2,815	16.67%	SD	3.34%	SD	N/A	No	None
22a	41	36	46	46	35,550	187.50%	-	1940.14%	-	-	-	-
22b	19	15	15	19	2,238	5.56%	-	-17.82%	-	-	-	-
Total	-	-	-	65	37,789	91.18%	PD	746.08%	PD	No	No	2 treated lesions.
23	15	17	13	17	1,704	-19.05%	SD	-54.49%	PR	No	No	Re-treated before 2nd f/u.
24a	7	7	4	7	109	-56.25%	-	-89.74%	-	-	-	-
24b	8	7	7	8	188	-42.86%	-	-82.72%	-	-	-	-
Total	-	-	-	15	297	-50.00%	PR	-86.19%	PR	No	No	2 treated lesions.
25	21	10	16	21	1,759	-38.24%	PR	-82.96%	PR	N/A	No	Re-treated before 2nd f/u.
26	[redacted]									Yes	No	Deceased prior to second follow-up.
27	62	54	56	62	98,168	-7.46%	SD	-28.32%	SD	No	No	None
28	33	30	33	33	17,106	-29.79%	SD	-4.26%	SD	No	No	Re-treated before 2nd f/u.
29	[redacted]									No	No	Deceased prior to second follow-up.
30a	19	20	17	20	3,685	17.65%	-	186.99%	-	-	-	-
30b	41	38	48	48	35,269	-15.79%	-	107.34%	-	-	-	-
Total	-	-	-	68	38,954	-8.11%	SD	112.93%	PD	No	No	2 treated lesions. Re-treated before 2nd f/u.
31	8	7	7	8	178	-33.33%	PR	-80.33%	PR	N/A	No	None
32	16	10	15	16	1,257	-23.81%	SD	-48.05%	PR	Yes	No	None
