# Supplementary material for: Pulsed Electric Field Ablation for Advanced Lung and Oligometastatic Disease: A Retrospective Study of 32 Consecutive Patients in a Community Hospital Setting
Source: Cancers (Basel). 2026 May 1;18(9):1459. doi: 10.3390/cancers18091459 (PMC13162631; doi:10.3390/cancers18091459)
Supplement: Supplementary file 1 [file cancers-18-01459-s001.zip › Table S2 - Supplemental Data Table - Markers.pdf]

Supplemental Data Table - Markers

Case	Age	Sex	Cancer Type	Cancer Stage	Molecular Markers	TMB (m/MB)	MSI status	3-Month Response per RECIST 1.1	3-Month Response per vRECIST	6-Month Response per RECIST 1.1	6-Month Response per vRECIST
1	72	Female	Squamous Cell Lung	IIIB	N/A	N/A	N/A	SD	SD	Deceased	Deceased
2	71	Female	Adenocarcinoma Lung	IV	KRAS; MAX, TP53	10.5	Stable	SD	PR	Deceased	Deceased
3	77	Female	Squamous Vaginal	IV	PD-L1+	7.9	Stable	SD	PR	PD	PD
4	69	Male	Squamous Cell Lung	IIIC	PD-L1+	N/A	Not Detected	PR	PR	Deceased	Deceased
5	86	Female	Breast Cancer, triple negative	IV	N/A	N/A	N/A	Deceased	Deceased	Deceased	Deceased
6	68	Male	B-Cell Lymphoma	IV	BCL6, CDK4, CHEK2; TP53, SPEN, RBM10	14.2	Stable	CR	CR	CR	CR
7	81	Male	Squamous Head and Neck	IV	PD-L1, DDX3X, KMT2D, also HPV 16 positive	2.1	Stable	SD	SD	SD	PR
8	88	Female	Renal Cell	IV	PD-L1 negative per Liquid Circulogene	N/A	N/A	SD	PR	SD	PR
9	76	Female	Adenocarcinoma Lung	IIB	N/A	N/A	N/A	SD	SD	SD	SD
10	67	Female	Adenocarcinoma Lung	IV	KRAS; TP53, AKT2, PTEN, DNMT3A	45	Stable	SD	SD	SD	SD
11	46	Male	Leiomyosarcoma, vaginal source	IV	PD-L1, TP53, TSC2, RB1	0	Stable	SD	SD	SD	PD
12	82	Female	Rectal	IV	PD-L1 negative per Liquid Circulogene	N/A	N/A	CR	CR	CR	CR
13	79	Male	Adenocarcinoma Lung	IV	PD-L1 TPS 0%. Liquid: CHEK2	0	Not Detected	SD	SD	SD	PR
14	77	Male	Adenocarcinoma Lung	IV	Liquid: PD-L1+, NTRK1	1	Not Detected	PD	PD	PR	PR
15	71	Male	Renal Cell, Papillary type	IV	N/A	N/A	N/A	SD	SD	SD	PD
16	85	Male	Melanoma	IV	BRAF, TP53, MAP2K1, NTRK1, SLX4. SMARCA4	83.8	Stable	PD	PD	Deceased	Deceased
17	67	Male	Renal Cell, Clear Cell	IV	KDM5C, VHL	3.9	Stable	PR	PR	SD	PR
18	68	Male	Squamous Head and Neck, HPV+	IV	FUBP1, FGF3, FGF4, PHGDH, RECQL4	3.2	Stable	SD	PD	PD	PD
19	68	Male	Adenocarcinoma Lung	IV	PIK3CA, STK11, MSH3, ARID2, RPL5, KEAP1, DNMT3A, MYC	11.6	Stable	Deceased	Deceased	Deceased	Deceased
20	86	Male	Squamous Head and Neck, HPV+	IV	PIK3CA, ZNF750, KMT2C, KLHL6	3.7	Stable	PR	PR	PD	PD
21	81	Male	Colon	IV	KRAS; APC, TCF7L2, MLH1, PMS2, MSH2, MSH6	13.2	Stable	SD	SD	SD	SD
22	87	Female	Urothelial Carcinoma	IV	TP53, DNMT3A, KDM6A, PTEN, RB1	3.9	Stable	PD	PD	PD	PD
23	80	Female	Large Cell Neuroendocrine	IV	PD-L1, KRAS, TP53, CDKN2A, TSC2, KEAP1, ARID1A, AKZF1, RBM10	7	Stable	PD	SD	SD	PR
24	49	Male	Carcinoid	I	Liquid Circulogene: PD-L1 +	N/A	N/A	PR	PR	PR	PR
25	80	Male	Carcinoid	I	N/A	N/A	N/A	SD	PR	PR	PR
26	86	Female	Adenocarcinoma Lung, acinar and papillary pattern	IV	EGFR, TP53, PTEN, CDKN2A	3.2	Stable	SD	PR	Deceased	Deceased
27	75	Male	Renal Cell	IV	Liquid: PD-L1+, VHL p.R161	2.6	Stable	SD	PR	SD	SD
28	75	Male	Adenocarcinoma Lung	IV	PD-L1, MDM2, MET, CDKN2A	2.6	Stable	SD	SD	SD	SD
29	83	Female	Renal Cell	IV	FANCF, VHL	3.9	Stable	PR	PR	Deceased	Deceased
30	66	Male	Colorectal Adenocarcinoma	IV	Mismatch repair retained. HER2 3+. No KRAS, NRAS, BRAF, KIT, PDGFRA.	3.7	Stable	SD	PD	SD	PD
31	32	Female	Adenocarcinoma Lung, mucinous features	IIIA	Liquid: PD-L1 +; ALK	0	Stable	SD	PD	PR	PR
32	81	Male	Renal Cell	IV	PD-L1 negative per Liquid Circulogene	0.8	Stable	SD	PR	SD	PR
